# Non‐coding RNA‐associated competitive endogenous RNA regulatory networks: Novel diagnostic and therapeutic opportunities for hepatocellular carcinoma

**DOI:** 10.1111/jcmm.17126

**Published:** 2021-12-14

**Authors:** Sattar Khashkhashi Moghadam, Babak Bakhshinejad, Ali Khalafizadeh, Bashdar Mahmud Hussen, Sadegh Babashah

**Affiliations:** ^1^ Research and Development Center of Biotechnology Tarbiat Modares University Tehran Iran; ^2^ Department of Molecular Genetics Faculty of Biological Sciences Tarbiat Modares University Tehran Iran; ^3^ Department of Pharmacognosy College of Pharmacy Hawler Medical University Erbil Kurdistan Region Iraq; ^4^ Center of Research and Strategic Studies Lebanese French University Erbil Kurdistan Region Iraq

**Keywords:** circular RNA, competing endogenous RNA networks, hepatocellular carcinoma, long non‐coding RNA, microRNA

## Abstract

Hepatocellular carcinoma (HCC), as the most prevalent liver malignancy, is annually diagnosed in more than half a million people worldwide. HCC is strongly associated with hepatitis B and C viral infections as well as alcohol abuse. Obesity and nonalcoholic fatty liver disease (NAFLD) also significantly enhance the risk of liver cancer. Despite recent improvements in therapeutic approaches, patients diagnosed in advanced stages show poor prognosis. Accumulating evidence provides support for the regulatory role of non‐coding RNAs (ncRNAs) in cancer. There are a variety of reports indicating the regulatory role of microRNAs (miRNAs) in different stages of HCC. Long non‐coding RNAs (LncRNAs) exert their effects by sponging miRNAs and controlling the expression of miRNA‐targeted genes. Circular RNAs (circRNAs) perform their biological functions by acting as transcriptional regulators, miRNA sponges and protein templates. Diverse studies have illustrated that dysregulation of competing endogenous RNA networks (ceRNETs) is remarkably correlated with HCC‐causing diseases such as chronic viral infections, nonalcoholic steatohepatitis and liver fibrosis/cirrhosis. The aim of the current article was to provide an overview of the role and molecular mechanisms underlying the function of ceRNETs that modulate the characteristics of HCC such as uncontrolled cell proliferation, resistance to cell death, metabolic reprogramming, immune escape, angiogenesis and metastasis. The current knowledge highlights the potential of these regulatory RNA molecules as novel diagnostic biomarkers and therapeutic targets in HCC.

**Abbreviations**
5mC: oxidize 5‐methylcytosine
5hmC: 5‐hydroxymethylcytosine
APC: adenomatous polyposis coli
CCA: cholangiocarcinoma
ceRNA: competing endogenous RNA
circRNA: circular RNAs
DLGAP1‐AS1: lncRNA DLGAP1 antisense 1
DUXAP8: double‐homeobox A pseudogene 8
EMT: epithelial‐mesenchymal transition
ERA: estrogen receptor alpha
FOXA1: FORKHEAD box A1
FOXM1: forkhead box protein M1
FUS: fused in sarcoma
GSN: gelsolin
HBC: hepatitis C
HBV: hepatitis B
HCC: hepatocellular carcinoma
LINC00160: long non‐coding RNA 00160
LncRNA: long ncRNA
MALAT1: metastasis‐associated lung adenocarcinoma transcript 1
MET: mesenchymal‐to‐epithelial transition
miRNA: MicroRNA
MRE: miRNA response element
NAFLD: nonalcoholic fatty liver disease
ncRNA: non‐coding RNAs
OLT: orthotopic liver transplantation
PARP: poly (ADP‐ribose) polymerases
PIK3R3: phosphoinositide‐3‐kinase regulatory subunit 3
piRNA: Piwi‐interacting RNA
PPM1F: protein phosphate, Mg2+ /Mn2+‐dependent 1F
Rb: retinoblastoma
RBP: RNA‐binding protein
ROCK2: Rho‐kinase 2
siRNA: small interfering RNA
SNAP23: synaptosome‐associated protein 23
snoRNA: small nucleolar RNA
snRNA: small nuclear RNA
SOX9: SRY‐box transcription factor 9
SOX9‐AS1: lncRNA SOX9 antisense RNA 1
TAM: tumour‐associated macrophages
TCF‐4: T‐cell factor‐4
TET: ten‐eleven translocation
TGF‐β: transforming growth factor‐beta
TRAIL: TNF‐related apoptosis‐inducing ligand
tRNA: transfer RNA
VAMP7: vesicle‐associated membrane protein 7
ZEB1: zinc finger E‐box‐binding homeobox 1
ZEB2: zinc finger E‐box‐binding homeobox 2

## 1 INTRODUCTION

### 1.1 Hepatocellular carcinoma

Hepatocellular carcinoma (HCC) accounts for an aggressive primary form of liver cancer. Annually, over 500,000 new cases of HCC are diagnosed across the world and its incidence continues to rise.^1^, ^2^ It is an epithelial tumour that originates from stem cells or mature hepatocytes characterized by chemotherapy resistance and poor prognosis.^3^ A multitude of genetic and epigenetic changes contributes to the multi‐step malignant transformation of liver tissue.^4^ Chronic viral infections of hepatitis B (HBV) and C (HCV), alcoholism and cirrhosis are recognized as the most important risk factors for HCC.^5^ The risk of disease development is also increased in chronic medical conditions such as diabetes mellitus and obesity. As the liver tissue plays a crucial role in glucose metabolism, diabetes mellitus can lead to a variety of liver‐associated disorders including chronic hepatitis, fatty liver, liver failure and cirrhosis.^6^ Orthotopic liver transplantation (OLT) and surgical resection are known as the most effective approaches for HCC treatment. However, a high rate of metastasis/recurrence (~50%–70%) has been observed within five years post‐operation.^7^ Although sorafenib and regorafenib are used as the first‐ and second‐line systemic chemotherapy for HCC, concerns about drug resistance, which leads to a high mortality rate, are rising. Over the recent decades, a large body of evidence has been obtained about the role played by genes crucial for cellular processes, such as cell cycle control, cell growth, apoptosis and migration in HCC development.^8^, ^9^, ^10^ This highlights the necessity of unravelling the mechanisms underlying HCC progression as well as finding efficient molecular biomarkers.

### 1.2 Non‐coding RNAs

Recent advances in transcriptome sequencing have revealed that less than 3% of human genome encodes exons, while almost 97% of genome is transcribed into non‐coding RNAs (ncRNAs) including microRNAs (miRNAs) and long non‐coding RNAs (lncRNAs).^11^, ^12^ NcRNAs are RNA molecules that do not code for proteins and play key roles in DNA replication, RNA splicing, translation and epigenetic regulation. Based on transcript length, ncRNAs are divided into two major categories: those shorter than ~200 nucleotides are known as short ncRNAs (miRNAs, piRNAs, snoRNAs, snRNAs, tRNAs) and those longer than ~200 nucleotides are known as long ncRNAs (lncRNAs, pseudogenes and circRNAs).^13^, ^14^, ^15^ The regulatory networks consisting of miRNA, lncRNA and mRNA have received attention in the study of biological mechanisms involved in cancer occurrence and progression.^16^ Now, the competing endogenous RNA (ceRNA) hypothesis, initially proposed by Salmena et al., is widely acknowledged by the scientific community.^17^ According to this hypothesis, a complex post‐transcriptional regulatory network mediated by miRNAs and sharing one or more miRNA response elements (MREs), protein‐coding RNAs and ncRNAs competes for binding to miRNAs. This leads to the modulated expression of different molecules in the network.^18^ There are mainly two cellular conditions needed for ceRNA to occur. Firstly, the relative concentration of ceRNAs and their miRNAs is important. Changes in the ceRNA expression levels need to be large enough to either overcome or relieve miRNA repression on competing ceRNAs. This is exemplified by RNA transcripts switched on or off at the transcriptional level in different developmental stages or physiological/pathological conditions. Secondly, the effectiveness of a ceRNA depends on the number of miRNAs it can sponge. This in turn depends on the accessibility of ceRNA to miRNA molecules, which is influenced by its subcellular localization as well as interaction with RNA‐binding proteins.^17^

### 1.3 MicroRNAs

miRNAs are short RNA strands with 18–23 nucleotides that regulate critical cellular processes. They are transcribed by RNA polymerase II or III as short RNA hairpin structures which are subsequently processed by the nuclear and cytoplasmic RNase III‐type enzymes.^19^ They act post‐transcriptionally via complementary base‐pairing with 3′‐untranslated region (3´‐UTR) of the target gene, but may also interact with 5′‐UTR and coding region.^20^, ^21^, ^22^ At least, 60% of human genes harbour target sites for miRNAs.^23^ miRNAs exert their effects through interaction between nucleotides 2–8 at their 5´‐ends (seed region) and mRNA target sites, leading to translational repression, cleavage or mRNA degradation.^24^ Regarding their function, miRNAs are frequently found in oncogenesis‐associated genomic regions. Therefore, they can be linked to relevant tumour properties, such as cell proliferation, apoptosis, differentiation and cell cycle regulation.^25^, ^26^, ^27^, ^28^, ^29^ A growing range of evidence has demonstrated the contribution of miRNAs to HCC‐related cellular processes and their potential use as prognostic and diagnostic markers.^10^ For instance, miR‐423 has been reported to play roles in HCC such as enhancing cellular invasiveness,^30^ contributing to tumorigenesis,^31^ cell cycle control, autophagy regulation,^32^ promoting cell growth and regulating the G1/S transition by targeting p21Cip1/Waf1.^33^ In another case, miR‐10b has been reported to exert its oncogenic role in HCC by targeting the expression of CUB and sushi multiple domains 1 (CSMD1).^34^ Also, miR‐92a has been proposed to contribute to tumour growth in HCC by targeting FBXW7.^35^

### 1.4 Long non‐coding RNAs

**FIGURE 1:**
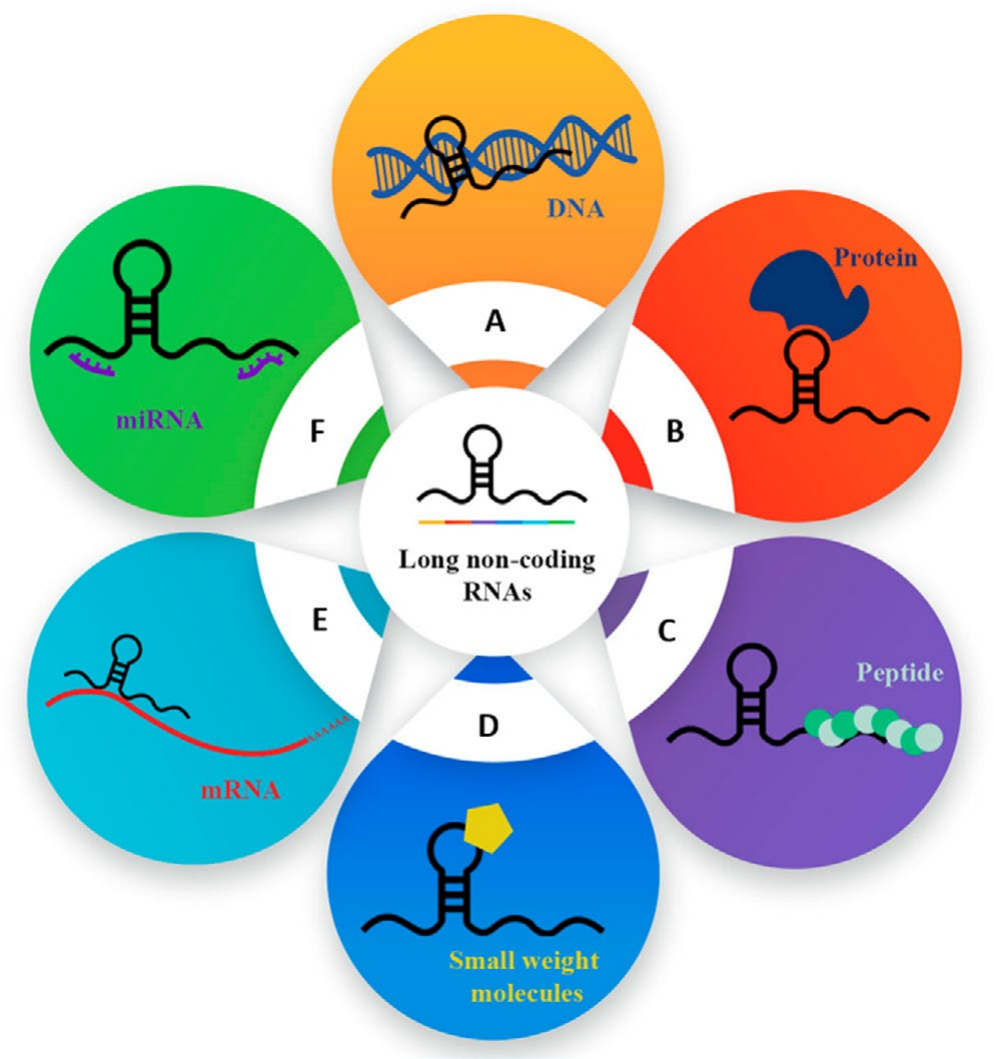
Different modes of action of long non‐coding RNAs. (A) LncRNA‐double‐stranded DNA interaction. (B) LncRNA‐protein interaction. (C) LncRNA‐peptide interaction. (D) LncRNA‐small‐weight‐molecule interaction. (E) LncRNA‐mRNA interaction. (F) LncRNA‐miRNA interaction

LncRNAs are RNA molecules with more than 200 nucleotides and have been known to be involved in tumorigenesis in a variety of cancer types.^36^, ^37^, ^38^ These molecules are transcribed by RNA polymerase ll from different regions of the target gene including enhancers (enhancer RNAs, eRNAs) and promoters (promoter upstream transcript, PROMPTS) and undergo post‐transcriptional processing events involving 5′‐end capping, 3′‐end polyadenylation and splicing.^39^, ^40^ LncRNAs are mostly located in the cytosol, where they target mRNAs and down‐regulate protein translation.^23^ They are heterogeneous molecules that perform various functions via interacting with DNA, RNA, proteins, peptides, small weight molecules, miRNAs and mRNAs (Figure 1). For instance, they regulate chromatin state and cell cycle, control mRNA stability, silence retrotransposons and competitively sponge miRNAs.^41^, ^42^, ^43^, ^44^ These regulatory RNAs play leading roles in the modulation of gene expression at epigenetic, transcriptional and post‐transcriptional levels, thereby contributing to a variety of cellular phenomena such as RNA processing, chromatin modification, apoptosis and invasion.^45^ Furthermore, some lncRNAs have been indicated to be abnormally expressed in human diseases, providing support for their involvement in pathogenesis. These functions underline the multi‐faceted role of lncRNAs in the regulation of gene expression. Another noteworthy point about lncRNAs is that they perform their specific functions by interacting with multiple proteins and hence regulating numerous cellular processes. Studies have demonstrated that lncRNAs can activate post‐transcriptional gene regulation, splicing and translation by binding to proteins. Therefore, determining possible lncRNA‐protein interactions (LPIs) is essential for unravelling lncRNA‐related activities.

### 1.5 Circular RNA

Circular RNAs (circRNA) are transcript isoforms generated from precursor mRNAs of protein‐coding genes in which back‐splicing between 3′ and 5′ splice sites forms a covalent circular structure. Compared with linear RNAs, circRNAs have a higher thermal stability and tissue specificity. Next‐generation RNA sequencing and bioinformatic analyses have revealed that circRNAs constitute a substantial fraction of eukaryotic transcriptome.^46^, ^47^, ^48^, ^49^, ^50^ CircRNAs perform various biological functions including miRNA sponging, interaction with RNA‐binding proteins (RBPs), translation of proteins and acting as mRNA translation brake. Due to the high structural stability of circRNAs, they have an inherent sponging capacity through which bind to miRNAs and prevent them from sequestering or suppressing their target mRNAs.^51^, ^52^, ^53^ For instance, SMARCA5 and circMTO1 sponge miR‐17‐3p and miR‐181b‐5p, respectively, thereby inhibiting the growth and metastasis of HCC cells.^54^

### 1.6 Exosomes

Exosomes are small endosomes extracellular vesicles with a size range of ~40 to 160 nm.^55^ Exosomes have gained attention over the past decade owing to their role as carriers of a wide range of biomolecules, including lipids, proteins, DNAs, mRNAs, lncRNA and miRNAs in both physiological and pathological processes. Hence, they illustrate a novel mode of intercellular communication and play a principal role in many cellular processes, such as immune response, signal transduction and antigen presentation. They represent potential use as biomarkers in a variety of cancers including HCC.^56^, ^57^ It has been demonstrated that exosomes can promote the occurrence and development of tumours and make a significant contribution to tumour invasion and metastasis.^58^ Exosomes can be divided into tumour and stromal cell secretions based on their origin in the tumour microenvironment.^59^ In a recent report, a urinary exosomal miRNA, miR‐532‐5p, has been suggested as a predictive biomarker for biochemical recurrence after radical prostatectomy (RP) in intermediate‐risk prostate cancer patients.^60^ Han et al^61^ have investigated the regulatory mechanism of exosomal lncRNA AFAP1‐AS1 in trastuzumab resistance of breast cancer. Based on the observations of this study, lncRNA AFAP1‐AS1 confers trastuzumab resistance through packaging into exosomes in breast cancer cells. AFAP1‐AS1 has also been shown to promote an AUF1‐mediated activation of ERBB2 translation, causing increased HER‐2 expression and trastuzumab resistance. Plenty of studies have reported the association of exosomal lncRNAs with HCC. For instance, Ma et al^62^ have proposed a mechanism in which exosomes derived from mesenchymal stem cells can transfer miR‐15a to HCC cells to inhibit the cellular proliferation, migration and invasion by negatively regulating SALL4.

## 2 COMPETING ENDOGENOUS RNA NETWORK (CERNA)

Here, we review the roles played by some of the important ceRNA regulatory networks in different steps of HCC development including cell growth and proliferation, metastasis, invasion, angiogenesis, apoptosis and chemoresistance. Different ceRNA networks and their mechanisms of action in HCC are summarized in Table 1.

**TABLE 1:** Different ceRNA interactions in hepatocellular carcinoma

LncRNA/CircRNA	Status	miRNA	Status	Deregulated protein	Mechanism of action	Ref.
LINC00160	Up‐regulation	miR‐132	Down‐regulation	PIK3R3	LINC00160 silencing suppresses the autophagy of HCC cells by decreasing the expression of *PIK3R3* via promotion of miR‐132 to inhibit drug resistance in HCC cells	70
MCM3AP‐AS1	Up‐regulation	miR‐194‐5p	Down‐regulation	FOXA1	MCM3AP‐AS1 promotes cell proliferation, colony formation, and cell cycle progression, and induces apoptosis.	81
MALAT1	Up‐regulation	miR‐140‐5p	Down‐regulation	Aurora‐A	MALAT1 knockdown in sorafenib‐resistant HCC cells increases their sensitivity to sorafenib treatment by enhancing Aurora‐A expression	86
AK002107	Up‐regulation	miR‐140‐5p	Down‐regulation	TGFBR1	AK002107 up‐regulates the expression of TGFBR1 to promote the proliferation, colony formation, and invasion of HCC	87
CASC2	Down‐regulation	miR‐24 and miR‐221	Up‐regulation	Caspase ‐3 and ‐8	CASC2 affects TRAIL resistance through indirectly targeting caspase‐3 and caspase‐8	88
DUXAP8	Up‐regulation	miR‐485‐5p	Down‐regulation	FOXM1	DUXAP8 facilitates HCC progression and resistance to PARP inhibitor via up‐regulating FOXM1	96
SOX9‐AS1	Up‐regulation	miR‐5590‐3p	Down‐regulation	SOX9	SOX9‐AS1 promotes HCC progression and metastasis through sponging miR‐5590‐3p	110
LINC00662	Up‐regulation	miR‐15a, miR‐16, and miR‐107	Down‐regulation	WNT3A	LINC00662 promotes HCC tumour growth and metastasis by activating Wnt/β‐catenin and up‐regulating WNT3A	115
LINC01352	Down‐regulation	miR‐135b	Up‐regulation	APC	LINC01352 suppresses tumour via decreasing the production of APC, and consequently activating Wnt/β‐catenin signalling	117
LINC01278	Up‐regulation	miR‐1258	Down‐regulation	Smad2, Smad3	LINC01278 down‐regulation reduces migration and invasion of HCC cells induced by β‐catenin and TGF‐β1	123
SNHG8	Up‐regulation	miR‐149‐5P	Down‐regulation	PPM1F	SNHG8 promotes HCC tumorigenesis and invasion by up‐regulating the expression of protein phosphatase, Mg2+/Mn2+‐dependent 1F	129
DLGAP1‐AS1	Up‐regulation	miR‐486‐5p	Down‐regulation	H3F3B	DLGAP1‐AS1 affects the proliferation of HCC cells by up‐regulating H3F3B	132
DLGAP1‐AS1	Up‐regulation	miR‐26a/b‐5p	Down‐regulation	IL‐6	DLGAP1‐AS1 promotes HCC tumorigenesis and EMT by involvement of IL‐6/JAK2/STAT3 and Wnt/β‐catenin pathways	136
DLX6‐AS1	Up‐regulation	miR‐15a‐5p	Down‐regulation	CXCL17	DLX6‐AS1 from HCC‐derived exosomes regulates CXCL17 through competitively binding to miR‐15a‐5p to induce M2 macrophage polarization, hence promoting HCC migration, invasion and EMT.	182
ZFPM2‐AS1	Up‐regulation	miR‐139	Down‐regulation	GDF10	ZFPM2‐AS1 promotes HCC cell proliferation and invasion through regulation of GDF10	139
CircSLC3A2	Up‐regulation	miR‐490‐3p	Down‐regulation	PPM1F	CircSLC3A2 promotes cell proliferation and invasion via up‐regulating PPM1F expression	54
CircTRIM33‐12	Down‐regulated	miR‐191	Up‐regulation	TET1	CircTRIM33–12 inhibits HCC cell proliferation, metastasis and immune evasion by up‐regulating TET1	143
CircGFRA1	Up‐regulated	miR‐498	Down‐regulation	NAP1L3	CircGFRA1 contributes to HCC progression by modulating miR‐498/NAP1L3 axis in HCC	148
CircC16orf62	Up‐regulated	miR‐138‐5p	Down‐regulation	PTK2	CircC16orf62 down‐regulation noticeably inhibits the expression level of PTK2 which further mediates AKT/mTOR signalling activation in HCC.	155
CircMTO1	Down‐regulated	miR‐9	Up‐regulation	P21	CircMTO1 inhibits HCC growth by up‐regulation of p21 via sponging miR‐9	160
CircSMG1.72	Up‐regulation	miR‐141‐3p	Down‐regulation	GSN	ERα can suppress HCC cell invasion through altering circRNA‐SMG1.72/miR‐141‐3p/GSN signalling	166

### 2.1 LncRNA/miRNA/mRNA networks

#### 2.1.1 LncRNA LINC00160/miR‐132/mRNA PIK3R3

Phosphoinositide 3‐kinase (*PI3K*) is a heterodimer consisting of a SH2‐containing regulatory subunit (p85) and a catalytic subunit (p110) with both subunits expressed in multiple isoforms.^63^ PI3K regulatory subunit 3 (*PIK3R3*) is one of the regulatory subunits of *PI3K* that elicits major effects on various cellular phenomena such as proliferation, differentiation, apoptosis and metabolism. *PIK3R3* can also regulate cell cycle by binding directly to retinoblastoma (Rb) protein through its N‐terminal 24 highly fidelity amino acids (N24).^64^ Long intergenic non‐protein‐coding RNA (LINC00160) has been reported to correlate with chemoresistance of breast cancer cells by regulating *TFF3* through the activity of the transcription factor C/EBPβ.^65^ miR‐132, a member of miR‐212/132 cluster, has been demonstrated to be dysregulated in several malignancies. The function of this miRNA is complicated. It can act as an oncogene in squamous cell carcinoma of the tongue or as a tumour suppressor in osteosarcoma, prostate, ovarian and non‐small‐cell lung cancers.^66^ Moreover, miR‐132 has been recognized as a biomarker in colorectal cancer (CRC) and it can inhibit the invasion and metastasis of CRC by targeting *ZEB2*.^67^ miR‐132 has also been found to play some roles in pancreatic cancer. Down‐regulation of this miRNA, through promoter methylation, can promote the progression and metastasis of pancreatic and prostate cancers.^68^, ^69^ There are multiple lines of reports supporting the role of miR‐132 in HCC. miR‐132 functions as a tumour suppressor in HCC by directly targeting *PIK3R3* and regulating the AKT/mTOR pathway. It can also suppress cell proliferation, colony formation, migration and invasion, as well as induce apoptosis in HCC cells.^66^ There is some evidence showing the relationship between LINC00160 and miR‐132 in HCC. It has been found that miR‐132 is down‐regulated in HCC compared with normal adjacent tissues and its overexpression directly targets *PIK3R3*, leading to inhibited cell proliferation, invasion and migration of HCC cells. Furthermore, LINC00160 silencing suppresses the autophagy of HCC cells by decreasing the expression of *PIK3R3* that is achieved by increasing the expression of miR‐132 to inhibit drug resistance in HCC cells.^70^

#### 2.1.2 LncRNA MCM3AP‐AS1/miR‐194/mRNA FOXA1

FORKHEAD box A1 (*FOXA1*) is a transcription factor, belonging to the forkhead box gene superfamily, that plays a crucial role in chromatin binding of transcription factors and is involved in the development of endoderm‐derived organs including pancreas, lung, liver and prostate.^71^, ^72^ *FOXA1* has been found to be able to bind to the promoters of more than one hundred genes correlated with the regulation of cell signalling and cell cycle.^73^ A range of evidence has demonstrated that *FOXA1* contributes to the development and progression of diverse types of malignancies including glioma, breast, stomach, lung, ovarian and oesophageal cancers. The function of this transcription factor might change based on the specific type of cancer.^74^, ^75^, ^76^, ^77^ *FOXA1* plays a growth inhibitory role, and its expression is correlated with markers of differentiation in prostate cancer.^73^ Also, it can act as a tumour suppressor in different types of cancer including breast, endometrium, bladder, liver and pancreas tumours.^72^ Similarly, MCM3AP‐AS1 is a lncRNA shown to be involved in various types of cancer. In line with this, MCM3AP‐AS1/miR‐211/KLF5/AGGF1 axis has been found to regulate angiogenesis in glioblastoma. Also, the overexpression of MCM3AP‐AS1 can promote lung cancer progression via regulating miR‐340‐5p/KPNA4 axis.^78^, ^79^, ^80^ The expression, clinical significance, functional role and underlying mechanism of MCM3AP‐AS1 have also been investigated in HCC. This lncRNA is up‐regulated in HCC, and its expression level is directly related to the tumour size as well as correlated with advanced tumour stage and poor prognosis of HCC patients. MCM3AP‐AS1 silencing has been shown to significantly up‐regulate miR‐194‐5p expression in HCC cells. In fact, MCM3AP‐AS1 acts as a molecular sponge for miR‐194‐5p by directly binding to complementary sequences, leading to increased expression of *FOXA1* as a target of miR‐194‐5p in HCC cells. Interestingly, *FOXA1* restoration is able to rescue MCM3AP‐AS1 knockdown‐induced proliferation inhibition, G1 arrest and apoptosis in HCC cells. These findings support a link between lncRNA MCM3AP‐AS1 and miR‐194‐5p/FOXA1 as well as provide evidence for the potential use of MCM3AP‐AS1 as a prognostic biomarker and therapeutic target in HCC.^81^

#### 2.1.3 LncRNA MALAT1/miR‐140/mRNA Aurora‐A

Metastasis‐associated lung adenocarcinoma transcript 1 (MALAT1) is a lncRNA with known pathogenic roles. The dysregulated expression of this lncRNA has been found to be correlated with clinical parameters and prognosis in several types of human cancers including HCC.^82^ A recent finding has revealed that MALAT1 is involved in temozolomide‐related chemoresistance in glioblastoma through inducing miR‐101 expression and regulating autophagy‐associated chemoresistance in gastric cancer via miR‐23b‐3p sequestration.^83^, ^84^, ^85^ Currently, sorafenib resistance is recognized as one of the primary obstacles of a successful chemotherapy in metastatic liver cancer. *Aurora*‐*A* is known as a tumour‐promoting molecule in HCC. It has been revealed that this lncRNA is involved in promoting sorafenib resistance in HCC cells and up‐regulated MALAT1 expression is strongly associated with down‐regulated miR‐140‐5p expression, increased *Aurora*‐*A* expression and poor outcomes in HCC patients. Additionally, MALAT1 is able to sponge miR‐140‐5p. Knockdown of MALAT1 in sorafenib‐resistant HCC cells has been shown to increase sensitivity to sorafenib treatment both *in vitro* and in *vivo*.^86^ These observations highlight the important role of MALAT1/miR‐140‐5p/Aurora‐A axis in sorafenib resistance and reinforce the notion that MALAT1 can be a prominent therapeutic target to overcome sorafenib resistance in HCC tumours.^87^

#### 2.1.4 lncRNA AK002107/miR‐140/mRNA TGFBR1

MiR‐140‐5p is a miRNA with dual roles. This miRNA has been shown to suppress the growth and metastasis of HCC cells by inhibiting *TGFBR1* and *FGF9* expression. TGFBR1 has been recognized as a promoter of cancer cell growth by inducing epithelial‐mesenchymal transition (EMT). The lncRNA AK002107 is involved in regulating *TGFBR1* through the modulation of miR‐140‐5p, leading to EMT in HCC. The silencing of this lncRNA inhibits HCC cell proliferation, colony formation and invasion. Consistent with these findings, the expression of AK002107 has been indicated to be up‐regulated in HCC compared with corresponding non‐cancerous tissues. The investigation of protein and RNA levels of *TGFBR1* following AK002107 knockdown and miR‐140‐5p inhibition confirmed that these two regulatory RNA molecules co‐ordinately regulate TGFBR1/EMT pathway in HCC cell lines. It has been found that silencing of AK002107 reduces *TGFBR1* expression and vimentin levels and increases E‐cadherin levels, resulting in EMT suppression. To sum up, AK002107 is markedly up‐regulated in HCC, competitively inhibits miR‐140‐5p and subsequently increases the expression of *TGFBR1*. These events finally promote the proliferation, colony formation and invasion of HCC cells. AK002107/miR‐140‐5p/TGFBR1/EMT pathway plays a critical role in tumorigenesis of HCC and offers potential use as a target for the development of novel diagnostic and therapeutic approaches against HCC.^87^

#### 2.1.5 LncRNA CASC2/miR‐24, miR‐221/mRNA TRAIL

TNF‐related apoptosis‐inducing ligand (*TRAIL*), as a member of *TNF* superfamily, can induce apoptosis in a variety of cancers including prostate, skin, thyroid, colon, kidney, pancreas, central nervous system, breast and haematological malignancies.^63^ *TRAIL* exerts its cellular effects through interaction with death receptors and the formation of downstream death‐inducing signalling complexes, leading to apoptosis‐causing activation of apical caspase‐3 and caspase‐8. It has been revealed that *TRAIL* resistance of HCC is associated with ncRNA regulation. miR‐24 and miR‐221 regulate the expression of caspase‐3 and caspase‐8, thereby affecting *TRAIL* resistance.^88^ The novel lncRNA susceptibility candidate 2 (CASC2) has been known to play roles in various human malignancies such as glioma and endometrial cancer.^89^ Based on the recent studies, CASC2 functions as a tumour‐suppressive lncRNA through a variety of mechanisms, for instance, sequestration of oncogenic miRNAs and repression of Wnt/β‐catenin signalling.^90^ CASC2 is also able to enhance the TRAIL resistance of HCC via regulating caspase‐3 and caspase‐8 expression by acting as a sponge for miR‐24 and miR‐221. These data lead us to the conclusion that CASC2 might contribute to enhancing *TRAIL* resistance in HCC and consequently promoting the treatment efficacy of TRAIL‐based therapies.^88^

#### 2.1.6 LncRNA DUXAP8/miR‐485/mRNA FOXM1

MiR‐485‐5p has been reported to act as a tumour suppressor in some cancers, including ovarian epithelial tumours and oral tongue squamous cell carcinoma. Some accumulating evidence supports the expression and role of this miRNA in HCC progression. It has been indicated that HCC cell proliferation, migration and invasion are suppressed and apoptosis is induced by up‐regulation of miR‐485‐5p and inhibition of *WBP2* protein expression to block the activation of Wnt/ β‐catenin signalling pathway.^91^ Poly (ADP‐ribose) polymerases (*PARP*) include a family of related enzymes that catalyse the post‐translational binding of poly (*ADP*‐*ribose*) to target proteins and play substantial roles in multiple cellular processes, such as modulation of chromatin structure, transcription, replication, recombination and DNA repair. Some PARP inhibitors (*PARPis*), such as olaparib, rucaparib and niraparib, have been approved by Food and Drug Administration (FDA) and the European Medicine Agency for the treatment of ovarian and breast cancers, particularly those bearing BRCA mutations.^92^ Forkhead box protein M1 (*FOXM1*) is a transcription factor of the Forkhead box (*Fox*) protein superfamily which shows overexpression in many different cancer types and is a regulator of cancer cell division, aggressiveness and metastasis. *FOXM1* activity has been found to boost all hallmarks of cancer, including enhanced cell proliferation, genome instability, angiogenesis and suppressed cell senescence.^93^, ^94^ Double‐homeobox A pseudogene 8 (*DUXAP8*) is a lncRNA that acts as regulatory factor in many cancers. For instance, *DUXAP8* has been shown to promote cell growth in renal carcinoma.^95^ Also, *DUXAP8* expression is up‐regulated in HCC and enhances the proliferation and invasion of HCC cells. The expression of this lncRNA is correlated with tumours of advanced grades, tumours with lymph node metastasis and patients with poor overall survival. *DUXAP8* can up‐regulate *FOXM1* by acting as a sponge for miR‐485‐5p and interacting with the RNA‐binding protein fused in sarcoma (FUS). These observations support the notion that targeting *DUXAP8* or up‐regulating miR‐485‐5p can inhibit HCC progression and increase sensitivity to *PARPis*. Therefore, it is insightful to assess the role of *DUXAP8*, miR‐485‐5p and *FOXO1* in the development of other types of cancer.^96^

#### 2.1.7 LncRNA SOX9‐AS1/miR‐5590/mRNA SOX9

SRY‐box transcription factor 9 (*SOX9*), as a member of *SOX* family, can keeps cells in undifferentiated state during development and is correlated with many signalling pathways such as *NOTCH*, transforming growth factor‐beta (TGF‐β)/Smad and Wnt/β‐catenin.^97^, ^98^ *SOX9* has been reported to be implicated in regulating various cellular processes including proliferation, apoptosis, migration, invasion, chemoresistance, autophagy, angiogenesis, immune escape and metastasis by controlling the transcription of a multitude of genes.^99^, ^100^ Multiple lines of evidence support the suggestion that *SOX9* is involved in the development of diverse types of malignancies such as bladder, brain, colon, cervical, gastric, endometrial, liver, and head and neck cancers.^101^, ^102^, ^103^, ^104^, ^105^, ^106^, ^107^, ^108^, ^109^ It has been revealed that *SOX9* is up‐regulated in HCC and contributes to the proliferation, migration and invasion of HCC cells. Similarly, the lncRNA SOX9 antisense RNA 1 (SOX9‐AS1) is up‐regulated in HCC and underlies HCC progression and metastasis. SOX9‐AS1 can sponge miR‐5590‐3p, leading to elevate SOX9 expression, and SOX9 in turn transcriptionally activates SOX9‐AS1. SOX‐9AS1 regulates EMT by regulating SOX9 and its known downstream Wnt/β‐catenin pathway. In conclusion, SOX9‐AS1/miR‐5590‐3p/SOX9‐positive feedback loop drives tumour growth and metastasis in HCC via Wnt/β‐catenin pathway. These findings highlight the potential of SOX9‐AS1 as a prognostic marker and treatment target in HCC.^110^

#### 2.1.8 LncRNA LINC00662/miR‐15, miR‐16 and miR‐107/mRNA WNT3A

Tumour‐associated macrophages (*TAM*) are the major components of tumour microenvironment that inhibit anti‐tumour immunity and promote tumour progression by expressing cytokines and chemokines.^111^ In line with this, *TAM*s have been indicated to be significant components of tumour microenvironment in HCC.^112^ It has been found that the tumour cell‐derived Wnt ligand stimulates M2 to transduce the polarization of *TAM*s through classical Wnt/β‐catenin signalling, which results in immunosuppression as well as blockade of Wnt secretion in tumour cells and/or activation of Wnt/β‐catenin signalling in TAMs. Thus, TAMs represent a potential tool for HCC treatment.^113^ It has been demonstrated that LINC00662 is up‐regulated in HCC and promotes HCC progression through both tumour cell‐ and macrophage‐dependent modes. These observations suggest the potential exploitation of LINC00662 as a prognostic biomarker in HCC patients.^114^ LINC00662 increases *WNT3A* expression and secretion by competitively binding to miR‐15a, miR‐16 and miR‐107. As a result of *WNT3A* secretion, LINC00662 activates Wnt/β‐catenin signalling in an autocrine manner and further promotes proliferation, cell cycle and invasion, while repressing apoptosis in HCC cells. By up‐regulating *WNT3A*, LINC 00662 can also activate Wnt/β‐catenin signalling in macrophages in a paracrine manner, which induces the polarization of M2 macrophages. These findings provide strong support for the contribution of LINC00662 to HCC tumour cell growth and metastasis. Therefore, LINC00662 holds considerable potential as a prognostic biomarker and therapeutic target in HCC.^115^

#### 2.1.9 LncRNA LINC01352/miR‐135/mRNA APC

Chronic hepatitis B virus (HBV) is responsible for at least half of HCC cases worldwide, and HBV infection plays a central role in hepatocarcinogenesis, particularly HCC.^116^ The lncRNA LINC01352 has been shown to have a regulatory role in many cancers and is involved in the initiation and progression of HBV‐related HCC. A recent study has investigated the interaction between HBx and LINC01352 in HBV‐related HCC. They have exhibited that this lncRNA, which is down‐regulated by HBx via the activity of estrogen receptor (*ERa)*, functions as a tumour suppressor and is associated with a better prognosis in HCC patients. Mechanistically, this lncRNA acts as a sponge for miR‐135b, leading to the decreased production of adenomatous polyposis coli (*APC*) and consequently activated Wnt/β‐catenin signalling. These lines of evidence highlight the pathogenesis of HBx in HCC and provide a basis for the identification of new therapeutic targets for this malignancy.^117^

#### 2.1.10 LncRNA LINC01278/miR‐1258/mRNA SMAD2, 3

*SMAD*s, a small family of structurally related proteins, provide a well‐known signalling effector pathway that is initiated by activated *TGF*‐*β* receptors. These proteins are signal transducers of *TGF*‐*β* family in organisms ranging from worms to human. In this pathway, TβRI serine/ threonine kinase phosphorylates *Smad2* and *Smad3* on terminal serine motif, enabling them to partner with *Smad4* and translocate to the nucleus, where they regulate the transcription of target genes.^118^, ^119^ Studies have revealed a range of clues on the role of miR‐1258 in diverse types of cancer. It has been indicated that *CKS1B* expression is negatively regulated by miR‐1258, which causes the inhibition of cell proliferation, migration and tumorigenicity in CRC cells, supporting the notion that miR‐1258 functions as a tumour suppressor in this malignancy.^120^ Another line of evidence has reported correlation between miR‐1258 and *E2F1*, demonstrating that overexpression of miR‐1258 inhibits breast cancer cell proliferation and blocks cell cycle in G0/G1 phase, while inducing apoptosis by down‐regulating *E2F1*.^121^ miR‐1258 is significantly down‐regulated in HCC. The overexpression of this miRNA significantly inhibits the growth, proliferation and tumorigenicity of liver cancer cells by increasing cell cycle arrest in G0/G1 phase and eliciting apoptosis.^122^ More importantly, the stable overexpression of miR‐1258 has been found to suppress cell migration and stemness, as well as enhance the sensitivity of HCC cells to chemotherapy drugs such as doxorubicin. In a recent report, luciferase assays have revealed the direct binding of miR‐1258 to *Smad2* and *Smad3*, thus attenuating TGF‐β/Smad signalling. Also, it has been shown that LINC01278 is a negative regulator of miR‐1258 and LINC01278‐mediated HCC metastasis is dependent on miR‐1258 expression. Moreover, miR‐1258 down‐regulation enhances LINC01278 expression. LINC01278 down‐regulation reduces migration and invasion of HCC cells induced by β‐catenin and TGF‐β1. Taken together, this study has discovered a novel mechanism for β‐catenin/TCF‐4‐LINC01278‐miR‐1258‐Smad2/3 feedback loop activation in HCC metastasis and provides support for the potential of LINC01278 as a therapeutic target in HCC.^123^

#### 2.1.11 LncRNA SNHG8/miR‐149/mRNA PPM1F

Small nucleolar RNAs (snoRNAs) are as a class of ncRNAs with 60–300 nucleotides. The lncRNA small nucleolar RNA host genes (*SNHG*s) are predominantly found in the nucleolus and most of them act as guide RNAs for the post‐transcriptional modification of ribosomal RNAs (rRNAs) and spliceosomal RNAs, and some are involved in the nucleolytic processing of the original rRNA transcripts.^124^ It has been reported that *SNHG*s are closely linked to tumour growth and metastasis. In line with this, *SNHG7* has been shown to drive breast cancer progression by acting as a sponge for miR‐381.^125^ Moreover, the role of these regulatory RNA molecules has been demonstrated in the malignant transformation of lung,^126^ gastric^127^ and colorectal^128^ cancers. The lncRNA SNHG8 sponges miR‐149‐5p to promote tumorigenesis and metastasis of HCC. The expression level of SNHG8 is significantly increased in HCC compared with the adjacent normal tissues, which provides an independent prognostic factor for tumour recurrence in HCC patients. Additionally, knockdown of SNHG8 has been shown to inhibit cell proliferation, invasion and lung metastasis *in vitro* and *in vivo*, while overexpression of this lncRNA reverses these effects. Mechanically, SNHG8 counteracts the tumour‐suppressive effects of miR‐149 in HCC cells by acting as a sponge for miR‐149‐5P. The expression of phosphatase, Mg2+/Mn2+‐dependent 1F, a target of miR‐149, is negatively associated with miR‐149 expression, but positively correlated with SNHG8 expression in HCC specimens. Based on these results, lncRNA SNHG8 promotes HCC tumorigenesis and invasion via sponging miR‐149 and acts as a prognostic factor of tumour recurrence in HCC patients. Also, it has potential application as a promising biomarker in HCC.^129^

#### 2.1.12 LncRNA DLGAP1 antisense 1/miR‐486/mRNA H3F3B

DLGAP1 antisense RNA 1 (*DLGAP1*‐*AS1*) is a lncRNA that is involved in colorectal cancer.^130^ According to recent findings, knockdown of *DLGAP1*‐*AS1* can repress CRC development and enhance sensitivity to 5‐fluorouracil (*5*‐*FU*) by modulating miR‐149‐5p/TGFB2/Smad2 signalling pathway *in vitro* and *in vivo*. Mechanistically, *DLGAP1*‐*AS1* acts as a sponge for miR‐149‐5p to regulate its function. Another study has revealed that *DLGAP1*‐*AS1* plays a central role in gastric cancer *in vitro* and *in vivo* by regulating miR‐628‐5p/AEG‐1 axis.^131^ A recent report has found the contribution of this lncRNA to cell proliferation in HCC. This study has shown that *DLGAP1*‐*AS1* is highly expressed in cancerous compared with normal tissues. Moreover, its knockdown significantly increases miR‐486‐5P levels and suppresses cell proliferation in HCC. The interaction between miR‐486‐5p and *DLGAP1*‐*AS1* occurs through the sponging mechanism. Also, it has been indicated that high expression of miR‐486‐5p leads to reduced cell proliferation and miR‐486‐5p suppression is able to offset the impact of DLGAP1‐AS1 silencing on HCC cell proliferation and apoptosis. *H3F3B* acts as a target of miR‐486‐5p and is positively regulated by *DLGAP1‐AS1* in HCC. Noteworthy, up‐regulation of *DLGAP1‐AS1* can partly revive the declined cell proliferation in response to *DLGAP1‐AS1* knockdown. In conclusion, these findings provide support for the notion that *DLGAP1‐AS1* plays an oncogenic part in HCC by acting as a sponge to modulate miR‐486‐5p/H3F3B axis and represents huge potential to serve as a prognostic biomarker and therapeutic target in HCC.^132^

#### 2.1.13 LncRNA DLGAP1‐AS1/miR‐26a/b/mRNA IL‐6

**FIGURE 2:**
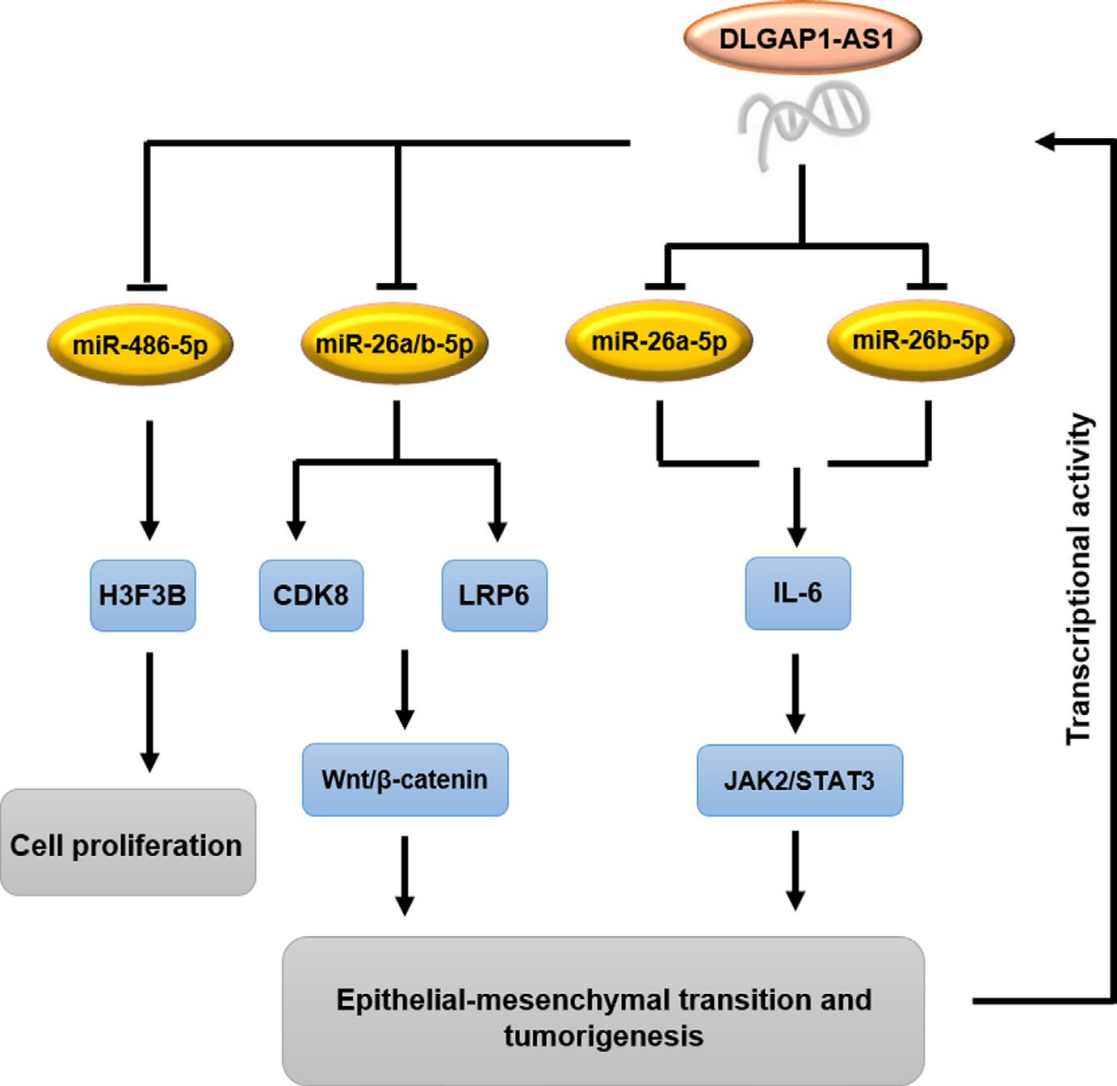
Molecular mechanism of relation between miR‐26a‐5p, miR‐26b‐5p and DLGAP1‐AS1: DLGAP1‐AS1, by acting as sponge for miR‐26a‐5p and miR‐26b‐5p, enhances the levels of an oncogenic cytokine IL‐6 that can activate JAK2/STAT3 signalling pathway and reciprocally elevate the transcriptional activity of DLGAP1‐AS1, thus forming a positive feedback loop. DLGAP1‐AS1 can also activate Wnt/β‐catenin pathway by positively regulating CDK8 and LRP6, downstream genes of miR‐26a/b‐5p. In this manner, DLGAP1‐AS1 contributes to HCC tumorigenesis and EMT by sponging miR‐26a‐5p and miR‐26b‐5p. On the contrary, DLGAP1‐AS1 knockdown significantly increases miR‐486‐5P levels and suppresses cell proliferation in HCC. The high expression of miR‐486‐5p leads to reduced cell proliferation, and miR‐486‐5p suppression is able to offset the impact of DLGAP1‐AS1 silencing on HCC cell proliferation and apoptosis. H3F3B acts as a target of miR‐486‐5p and is positively regulated by DLGAP1‐AS1 in HCC. The up‐regulation of DLGAP1‐AS1 can partly revive the declined cell proliferation in response to DLGAP1‐AS1 knockdown

Some recent studies have focused on the role of *DLGAP1*‐*AS1* in metastasis and attempted to unravel the mechanisms through which this lncRNA exerts effects on EMT. EMT is an important cellular programme that occurs during embryogenesis, tissue regeneration, organ fibrosis and wound healing. EMT is a trans‐differentiation process during which epithelial cells incrementally lose their cobblestone epithelial appearance in monolayer cultures to adopt a spindle‐shaped, mesenchymal morphology. EMT is also reversible via a process which is called mesenchymal‐to‐epithelial transition (MET). In MET, mesenchymal cells revert back to an epithelial state.^133^, ^134^ A large body of accumulating evidence has implied the fact that cells lose their polarity and cell‐cell adhesion to achieve invasive and migratory properties. Accordingly, EMT is associated with tumour progression and metastasis.^99^ Numerous studies have demonstrated the role of miRNAs in EMT regulation. In line with this, the regulatory role of miR‐1976 has been shown in EMT and cancer stem cell of breast cancer.^135^ *DLGAP1*‐*AS1* has been found to be up‐regulated in HCC cells and capable of driving HCC progression and EMT. This lncRNA sequesters the HCC‐inhibitory miRNAs, miR‐26a‐5p and miR‐26b‐5p, by acting as a sponge. This leads to the enhanced levels of an oncogenic cytokine IL‐6 that can activate *JAK2*/*STAT3* signalling pathway and reciprocally elevate the transcriptional activity of *DLGAP1*‐*AS1*, thus forming a positive feedback loop. Moreover, it has been shown that cancer‐promoting effects of *DLGAP1*‐*AS1* in HCC cells can happen through activating Wnt/β‐catenin pathway by positively regulating *CDK8* and *LRP6*, downstream genes of miR‐26a/b‐5p. Based on this, *DLGAP1*‐*AS1* contributes to HCC tumorigenesis and EMT by sponging miR‐26a‐5p and miR‐26b‐5p. IL‐6/JAK2/STAT3 and Wnt/β‐catenin pathways play crucial roles in mediating the oncogenic function of *DLGAP1*‐*AS1* (Figure 2). These lines of evidence suggest the potential of *DLGAP1*‐*AS1* for HCC treatment.^136^

#### 2.1.14 LncRNA ZFPM2‐AS1/miR‐139/ mRNA GDF10

GDF10 is a member of transforming growth factor‐beta (TGF‐β) superfamily that plays an important role in cell proliferation and differentiation. It is also known as BMP‐3B due to its close relationship with bone morphogenetic protein‐3 (BMP3), another member of the TGF‐β superfamily.^137^ miR‐139 mainly functions as a tumour suppressor in HCC. It can suppress the proliferation, migration and invasion of HCC cells and induce HCC cell apoptosis via down‐regulating a number of target genes, such as T‐cell factor‐4 (TCF‐4), Rho‐kinase 2 (ROCK2), zinc finger E‐box‐binding homeobox 1(ZEB1) and 2 (ZEB2).^138^ The lncRNA ZFPM2‐AS1 is up‐regulated in HCC. Silencing of ZFPM2‐AS1 inhibits cell proliferation, migration and invasion and promotes cell apoptosis in vitro. Mechanistically, lncRNA ZFPM2‐AS1 can bind to miR‐139 as a ceRNA and release the binding of miR‐139 to GDF10, hence regulating the expression of GDF10 at the post‐transcriptional level. In conclusion, lncRNA ZFPM2‐AS1 can act as an oncogene to induce HCC cell proliferation, invasion and metastasis, and the mechanism is mediated by ZFPM2‐AS1/miR‐139/GDF10 axis. It has been suggested that ZFPM2‐AS1 can serve as a prognostic biomarker for HCC patients.^139^

### 2.2 Circular RNA/miRNA/mRNA networks

#### 2.2.1 CircRNA TRIM33–12/miR‐191/mRNA TET1

DNA demethylation is a highly regulated process that is mediated by the ten‐eleven translocation (TET) family of dioxygenases. The TET enzymes (including TET1, TET2 and TET3) oxidize 5‐methylcytosine (5mC) into 5‐hydroxymethylcytosine (5hmC) to initiate the process of DNA demethylation.^140^, ^141^ It has been reported that 5hmC/TET1 expression is correlated with HCC progression. This indicates that DNA methylation/demethylation may be regulated by the TET family of methylcytosine dioxygenases.^142^ Zhang et al analysed the role of tripartite motif‐containing 33 (TRIM33)‐derived circRNAs and their effects on miR‐191 in HCC. They reported that the expression levels of circ‐TRIM33–12 were remarkably decreased in HCC tissues compared with adjacent normal ones. Also, they found that circ‐TRIM33–12 functionally and mechanistically inhibits HCC metastasis, proliferation and immune evasion by sponging miR‐191 and up‐regulating TET1 expression, highlighting its tumour‐suppressive role in HCC progression. Hence, circ‐TRIM33–12 can serve as a therapeutic target for HCC patients.^143^

#### 2.2.2 CircRNA GFRA1/miR‐498/mRNA NAP1L3

The nucleosome assembly proteins (NAP) represent a family of evolutionarily conserved histone chaperones consisting of five members in mammals. They were first recognized in mammalian cells and play crucial roles in maintaining cell viability, particularly in the formation and maintenance of the nervous system.^144^ miR‐498 has been indicated to be abnormally expressed in several types of human cancers. For instance, miR‐498 is down‐regulated in colorectal,^145^ ovarian^146^ and oesophageal cancer.^147^ CircRNA GFRA1 is dysregulated in many cancers and acts as predictive marker for various types of malignancies. It has shown that circGFRA1 expression is significantly increased in HCC tissues and cells. The expression of circGFRA1 is negatively correlated with the expression of miR‐498, but a positive correlation has been found between circGFRA1 and NAP1L3 expression in HCC tissues. Moreover, silencing circGFRA1 has been demonstrated to inhibit the growth and invasion of HCC. Mechanistically, circGFRA1 might exert its effects by sponging miR‐498. miR‐498 overexpression or NAP1L3 inhibition can abrogate the oncogene role of circGFRA1 in HCC. In conclusion, findings show that circGFRA1 can contribute to HCC progression by modulating the miR‐498/NAP1L3 axis in HCC.^148^

#### 2.2.3 CircRNA C16orf62/miR‐138‐5p/mRNA PTK2

Protein tyrosine kinase 2 (PTK2) is a member of the non‐receptor protein tyrosine kinase family that regulates cell survival, proliferation, migration, invasion and adhesion via scaffolding and kinase activity. PTK2 expression has been explored in several human epithelial malignancies including breast, ovarian, colorectal and lung cancers.^149^, ^150^ PTK2 is overexpressed in HCC and its overexpression is correlated with expression of liver cancer stem cell (CSC) genes, recurrence and poor patient survival. PTK2 functionally stimulates the Wnt/β‐catenin pathway by increasing the number of CSC subpopulations in HCC. In this manner, PTK2 enhances tumorigenicity and sorafenib resistance in HCC.^151^ The AKT serine/threonine kinase is an oncogenic protein that regulates cell survival, proliferation, growth, apoptosis and glycogen metabolism. It has been indicated that overexpression of phosphorylated AKT can be considered as a therapeutic target for treating malignant tumours such as breast cancer.^152^ AKT pathway plays an important role in the regulation of several processes involved in the development and progression of HCC; such as controlling growth, proliferation and survival of tumour cells.^153^ miR‐138‐5 has been reported to act as a tumour suppressor in several types of human cancers.^154^ The function and molecular mechanism of circC16orf62 have been investigated in HCC. CircC16orf62 has been shown to be significantly up‐regulated in HCC. It can promote proliferation, metastasis and aerobic glycolysis in HCC. CircC16orf62 has been demonstrated to act as a molecular sponge for miR‐138‐5p and is a competitive endogenous RNA for PTK2, promoting AKT/mTOR pathway activation. Hence, circC16orf62 functions as an oncogene in HCC progression and behaves as a competitive endogenous RNA for miR‐138‐5p binding, thus activating the AKT/mTOR pathway. Based on these findings, circC16orf62 is an oncogene that acts through miR‐138‐5p/PTK2/Akt axis in HCC cells. This shows the potential of circC16orf62 as a therapeutic target in HCC patients.^155^

#### 2.2.4 CircRNA MTO1/miR‐9/mRNA p21

One of the main engines that drives cellular transformation is the loss of proper control of the mammalian cell cycle. p21, a well‐established cyclin‐dependent kinase (cdk) inhibitor, was found to function as a cell cycle inhibitor and anti‐proliferative effector in normal cells and is dysregulated in some cancers. It has been suggested that p21 can act as a tumour suppressor in brain, lung and colon cancers.^156^, ^157^ miR‐9 is implicated in the regulation of a variety of tumours such as glioma^158^ and breast cancer.^159^ Han et al have investigated the correlation of circMTO1, miR‐9 and p21 in HCC cells. Based on their results, circMTO1 is down‐regulated in HCC tissues. Functionally and mechanistically, circMTO1 inhibits HCC growth by sponge activity on miR‐9 and up‐regulation of p21 expression, demonstrating its tumour‐suppressive role in HCC development. Moreover, the *in vivo* intervention of circMTO1 illustrates its potential in HCC therapy. In conclusion, their results indicate that circMTO1 can serve as a predictive and therapeutic target for HCC patients.^160^

#### 2.2.5 ERA/circRNA‐SMG1.72/miR‐141‐3p/mRNA GSN

Suppressor with morphogenetic effect on genitalia (*SMG1*) is a member of phosphatidylinositol 3‐kinase‐related protein kinases (*PIKK*s).^161^, ^162^ *SMG1* has a well‐known role in nonsense‐mediated decay (*NMD*), which is responsible for the degradation of mRNAs containing premature termination codons and has also been reported to be implicated in the regulation of DNA damage responses, oxidative and hypoxic stress responses, telomere maintenance and stress granule formation. *SMG1* has been found to suppress tumour growth by regulation of both *p53* and *Cdc25A* signalling pathways.^163^ In HCC, *SGM*‐*1* is down‐regulated^161^ and its abnormal expression is markedly associated with differentiation, clinical stage and serum AFP levels, suggesting that this protein might be involved in the pathogenesis and development of HCC. A regulatory role for steroid hormones in hepatic malignant transformation has been suggested after a consistent gender disproportion was observed in the incidence of HCC worldwide.^164^ The expression of estrogen receptors (*ER*s) and their variants plays a crucial role in hepatocarcinogenesis and is correlated with the male prevalence of HCC as well as specific viral infections.^165^ Recently, it has been shown that *ERA* can reduce HCC cell invasion by suppressing circSMG1.72, which occurs via transcriptional regulation via directly binding to the 5′ promoter region of its host gene SMG1. Additionally, *ERA*‐suppressed circSMG1.72 is able to sponge and inhibit the expression of miR‐141‐3p, which leads to the increased translation of gelsolin *(GSN)* mRNA through reduced miRNA binding to its 3´‐UTR. Altogether, these findings provide support for the notion that *ERA* can suppress HCC cell invasion via altering ERA/circRNA‐SMG1.72/miR‐141‐3p/GSN axis and this signalling pathway can be considered as a target for suppression of HCC progression.^166^

#### 2.2.6 CircRNA SLC3A2/miR‐490‐3p/mRNA PPM1F

Protein phosphate, Mg2+ /Mn2+‐dependent 1F (*PPM1F*) acts as a regulator of apoptosis, cell proliferation and metastasis in a variety of cancer cells. It facilitates cell motility and invasiveness as well as represses apoptosis via modulating TAK1‐IKK‐NF‐κB pathway.^54^ It has been found that *PPM1F* expression is correlated with smoking behaviour. Also, it shows high expression in early and advanced (Stages 3–4) stages of breast cancer. *PPM1F* functions as a phosphatase to dephosphorylate p53, leading to p53 inactivation and degradation. *PPM1F* expression has been shown to be correlated with A9‐nAChR expression. *PPM1F* can function downstream of A9‐nAchR to amplify nicotine‐induced carcinogenic signals. Thus, the expression of this molecule represents potential use for prognosis, diagnosis and treatment of cancer.^167^ In gastric tumour samples, *PPM1F* has been shown to be down‐regulated, but miR‐590 is up‐regulated and the expression levels of both molecules are associated with tumour recurrence. In addition, miR590 plays an oncogenic role through targeting *PPM1F* and acts as a prognostic factor for tumour recurrence.^168^ In HCC patients, *PPM1F* is up‐regulated and functions as a prognostic factor of poor survival. *PPM1F* up‐regulation has been suggested to be caused by binding of six possible miRNA (miR‐490‐3P, miR‐186‐5P, miR‐200b‐P, miR200c‐3P, miR‐425‐5P and miR‐429). miR‐490‐3p down‐regulation is significantly correlated with *PPM1F* up‐regulation. The low expression of this miRNA is also associated with poor survival and tumour recurrence in HCC patients. These findings provide evidence for the suppressive effects of miR‐490‐3p on HCC cell proliferation and invasion by targeting *PPM1F*. It has also been revealed that circRNA SLC3A2 is up‐regulated in HCC tissues and stimulates cell proliferation and invasion by sponging miR‐490‐3p and up‐regulating *PPM1F* expression, suggesting that circSLC3A2 might function as an oncogenic factor in HCC through modulation of miR‐490‐3p/PPM1F axis. These data suggest that circRNA SLC3A2 represents potential use as a biomarker for the diagnosis and treatment of HCC.^54^

### 2.3 The contribution of exosomal ncRNA to HCC pathogenesis

#### 2.3.1 LINC00511/exosome secretion/invadopodia

Invadopodia are actin‐rich protrusions of the plasma membrane that play key roles in process of tumour metastasis.^169^ Studies have revealed the mechanism by which the abnormal expression of lncRNAs affects exosome secretion in tumour cells. LINC00511 is an oncogene that plays a negative regulatory role in cell proliferation, apoptosis, invasion, cell cycle, progression migration, metastasis and chemoresistance. It is overexpressed in diverse types of malignancies including breast, lung and liver cancers.^170^, ^171^, ^172^ There is evidence showing the correlation between LINC00511 exosome secretion and invadopodia formation in HCC. The process of tumorigenesis is associated with a remarkable increase in vesicle secretion in HCC. Also, the expression of LINC00511 is significantly increased in HCC tissues. Abnormally expressed LINC00511 induces invadopodia formation in via regulating the colocalization of vesicle‐associated membrane protein 7 (VAMP7) and synaptosome‐associated protein 23 (SNAP23) to induce formation of invadopodia which are key secretion sites for MVBs and controlling exosome secretion. According to these observations, LINC00511‐induced invadopodia formation supports ECM degradation and tumour invasion. Also, LINC00511 induces the release of exosomes and promotes tumour progression.^173^

#### 2.3.2 LncRNA DLX6‐AS1/miR‐15a‐5p/mRNA CXCL17

Macrophages are crucial innate cells of immune system that have plenty of physiological functions. Tumour‐associated macrophages (TAMs) exist in the cancer microenvironment and affect the growth, development and metastasis of cancers through interacting with cancer cells.^174^ Macrophages can be polarized into classically activated M1 macrophages or alternatively activated M2 macrophages.^175^ Moreover, the interaction of polarized macrophages with cancer cells plays an essential role in many cancer types including HCC. Distal‐less homeobox 6 antisense 1 (DLX6‐AS1) is located in the 7q21.3 chromosomal region in humans and found to be overexpressed as an oncogenic LncRNA^176^ in a variety of tumour tissues such as gastric^177^ and colorectal cancers.^178^ DLX6‐AS1 has been demonstrated to function in tumours by ceRNA for binding to and inhibiting the function of miRNAs. This lncRNA plays a crucial role in a range of biological processes, such as regulating tumour proliferation, migration and invasion.^179^ Tumour‐derived exosomes can be ingested by macrophages in the tumour microenvironment and eventually promote tumour progression and metastasis.^180^ There are some reports on the functions of HCC‐derived exosomes in human cancers. For example, increased migration, invasion and EMT, as well as reduced E‐cadherin and elevated vimentin levels, have been detected in HCC cells co‐cultured with HCC‐derived exosomes.^181^ The oncogenic role of DLX6‐AS1 in HCC‐derived exosomes occurs through M2 macrophage polarization and miR‐15a‐5p/C‐X‐C motif chemokine ligand 17 (CXCL17) axis. DLX6‐AS1 inhibits miR‐15a‐5p, leading to promotion of M2 macrophage polarization to stimulate the invasion and metastasis of HCC. CXCL17 silencing can reduce the ability of migration, invasion and EMT. In conclusion, DLX6‐AS1 in HCC‐derived exosomes regulates CXCL17 through competitively binding to miR‐15a‐5p to induce M2 macrophage polarization, hence promoting HCC migration, invasion and EMT.^182^

#### 2.3.3 LncRNA DANCR/HCV‐HCC

Differentiation antagonizing non‐protein‐coding RNA (DANCR) is an 855‐nucleotide lncRNA located on the human chromosome 4q12 and was first identified as a suppressor during epidermal progenitor cell differentiation.^183^ Recently, studies have proposed that DANCR can act as an oncogene in diverse tumour types such as gastric^184^ and prostatic cancers.^185^ It is considered as a tumour inhibitor in breast cancer through degrading the epigenetic tumour regulator EZH2. In gliomas, DANCR modulates growth and metastasis by targeting the miR‐216a/LGR5 axis and PI3K/AKT signalling pathway.^186^ Furthermore, it plays roles in cholangiocarcinoma (CCA). DANCR up‐regulation can promote CCA progression through transcriptional inactivation of the target tumour suppressor gene FBP1.^187^ DANCR functions as a ceRNA to modulate HCC proliferation and metastasis by interfering with miR‐27a‐3p in the ROCK1/LIMK1/COFILIN1 pathway.^188^ Wang et al identified the role of circulating exosomal DANCR in hepatitis C virus‐related hepatocarcinogenesis. Based on their results, DANCR is up‐regulated following hepatitis C virus infection and recognized as the lncRNA most relevant to hepatitis C virus‐related HCC in tumour tissues. In addition, the expression level of circulating exosomal DANCR has been shown to be positively associated with HCC recurrence. The lncRNA DANCR is highly relevant to the progression of HCV‐HCC, and circulating exosomal DANCR might serve as a non‐invasive prognostic biomarker for HCV‐HCC prognosis.^189^

## 3 CONCLUSION AND PERSPECTIVE

**FIGURE 3:**
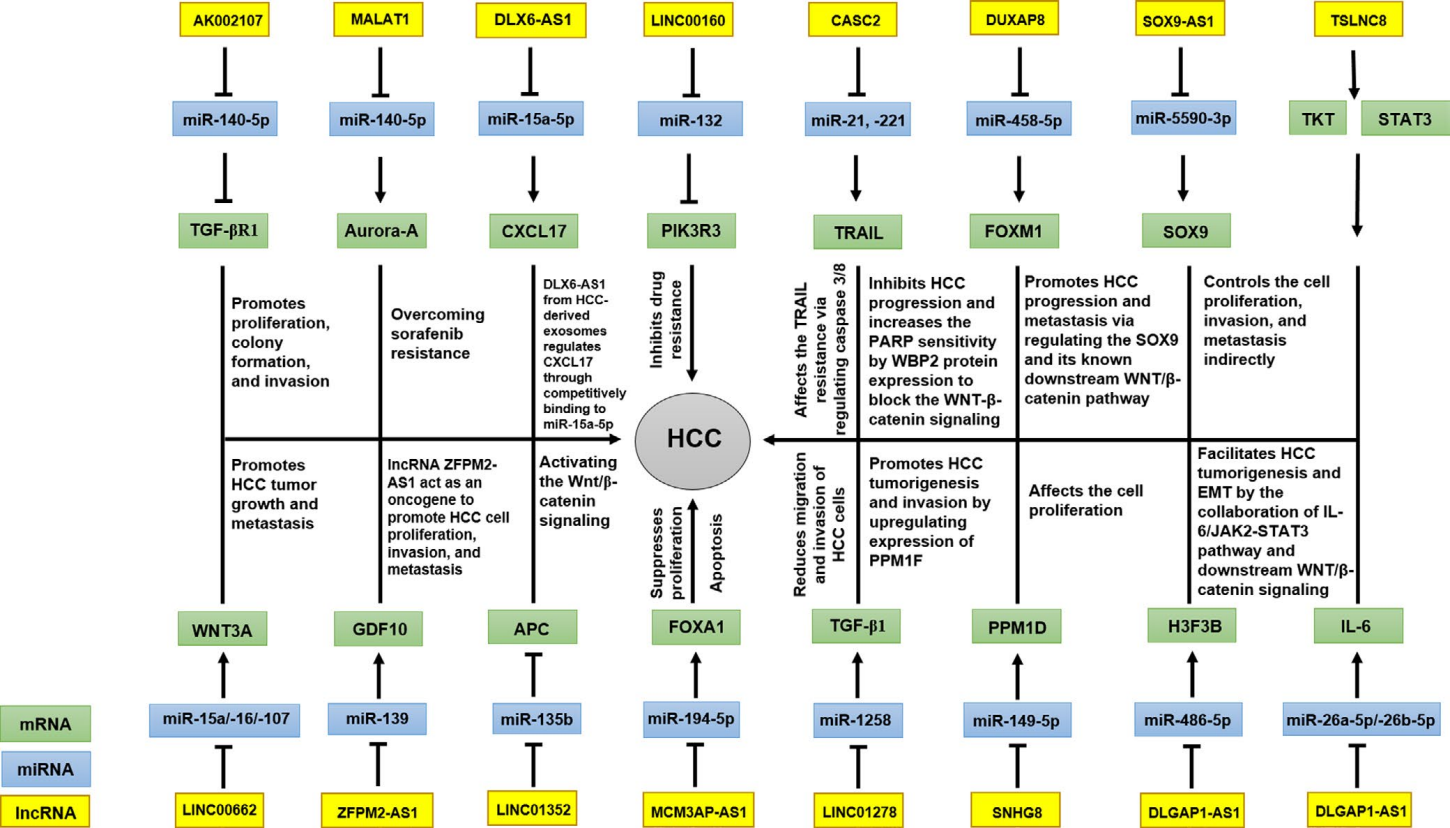
ceRNET and their pathways in HCC. The diagram demonstrates relation among lncRNAs, miRNAs and mRNAs as well as their molecular mechanisms in HCC

**FIGURE 4:**
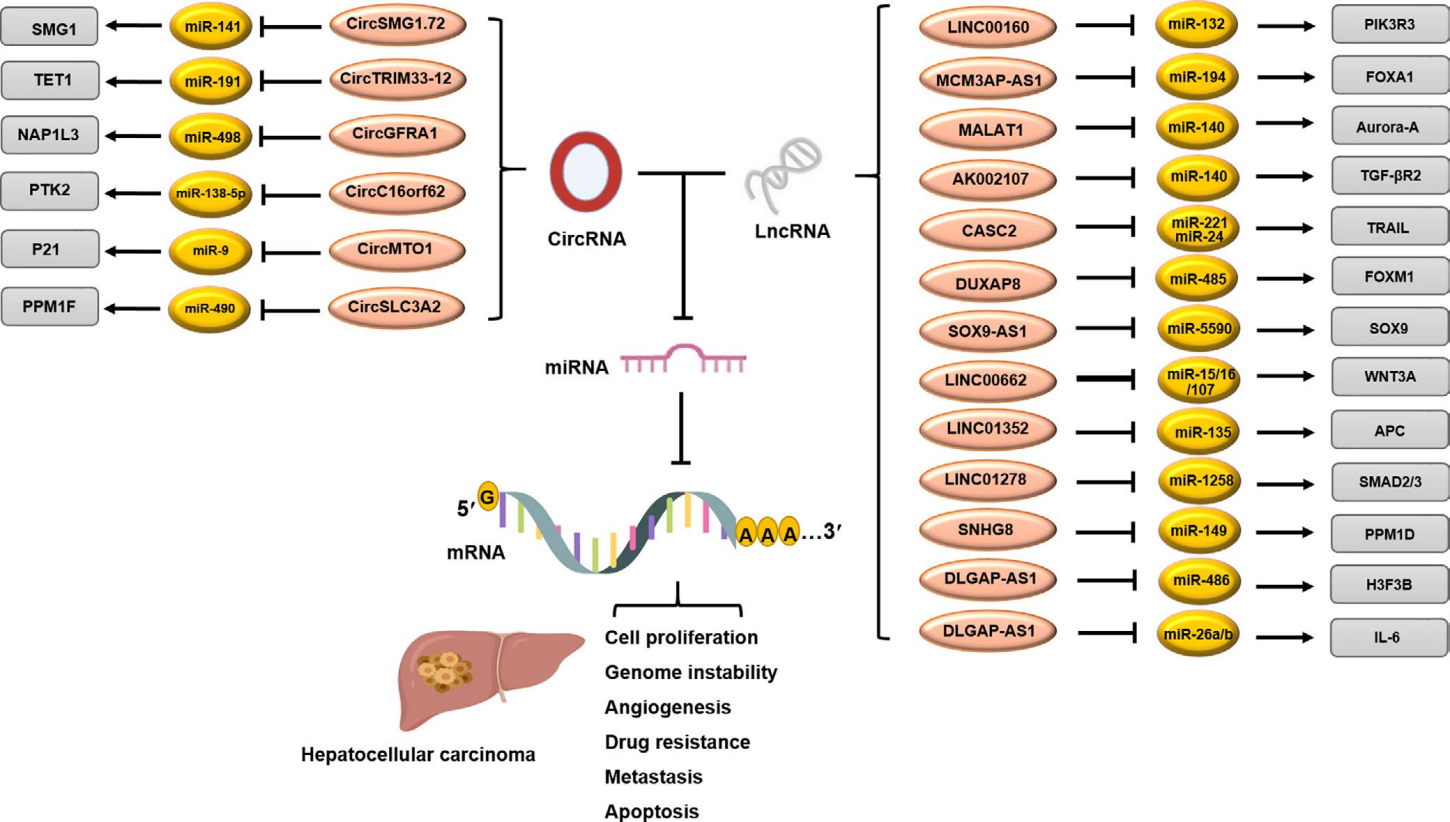
LncRNA and circRNA regulate miRNA expression by acting as sponge and consequently affecting the hallmarks of hepatocellular carcinoma

Since the discovery of the first ceRNAs, plenty of studies have demonstrated that RNA molecules that compete for shared target RNAs are important components of gene regulation in many metabolic and other cell‐related processes. Different ncRNAs can act as ceRNA and modulate the expression of mRNAs via sponging mechanism (Figures 3 and 4). Nonetheless, it should be noted that the ceRNA hypothesis is still being debated due to the fact that most experimental evidence is based on expression profiling such as qRT‐PCR. Furthermore, studies that have modelled transcriptome‐wide miRNA target site abundance suggest that physiological changes in the expression levels of most individual transcripts, including lncRNAs, are insufficient to modulate miRNA activity.^190^, ^191^, ^192^ Thus, further research is needed to establish the miRNA sponge (ceRNA) mechanism for lncRNAs. Accumulating findings have provided support for the contribution of ceRNETs to the initiation and development of malignant tumours. Deciphering the molecular mechanisms associated with ceRNET in HCC allows us to obtain insights into the potential of lncRNAs and circRNAs as predictive, prognostic and diagnostic biomarkers for this malignancy. A range of evidence has indicated the involvement of dysregulated ceRNETs in various characteristics of HCC including tumour initiation, cell proliferation and growth, progression, metastasis, EMT, apoptosis and angiogenesis. Understanding the role and mechanism of action of ceRNAs in malignant transformation of HCC cells can shed light on the development of new RNA‐based diagnostic and therapeutic strategies. The contribution of lncRNAs and miRNAs to HCC development demonstrates the feasibility of manipulating the expression levels of these molecules as an efficient diagnostic and therapeutic strategy. However, further investigation is required to pave the way for using these findings in the clinic.

## CONFLICT OF INTEREST

The authors report no competing interest.

## AUTHOR CONTRIBUTION

**Sattar Khashkhashi Moghadam:** Investigation (equal); Validation (equal); Visualization (equal); Writing – original draft (equal); Writing – review & editing (equal). **Babak Bakhshinejad:** Writing – original draft (equal); Writing – review & editing (equal). **Ali Khalafizadeh:** Visualization (equal); Writing – original draft (equal). **Bashdar Mahmud Hussen:** Visualization (equal); Writing – original draft (equal); Writing – review & editing (equal). **Sadegh Babashah:** Conceptualization (lead); Project administration (lead); Supervision (lead); Writing – review & editing (equal).
